# Yeast as a system for modeling mitochondrial disease mechanisms and discovering therapies

**DOI:** 10.1242/dmm.020438

**Published:** 2015-06-01

**Authors:** Jean-Paul Lasserre, Alain Dautant, Raeka S. Aiyar, Roza Kucharczyk, Annie Glatigny, Déborah Tribouillard-Tanvier, Joanna Rytka, Marc Blondel, Natalia Skoczen, Pascal Reynier, Laras Pitayu, Agnès Rötig, Agnès Delahodde, Lars M. Steinmetz, Geneviève Dujardin, Vincent Procaccio, Jean-Paul di Rago

**Affiliations:** ^1^ University Bordeaux-CNRS, IBGC, UMR 5095, 1 rue Camille Saint-Saëns, Bordeaux F-33000, France; ^2^ European Molecular Biology Laboratory (EMBL), Genome Biology Unit, Meyerhofstrasse 1, Heidelberg 69117, Germany; ^3^ Department of Genetics, Institute of Biochemistry and Biophysics, Polish Academy of Sciences, Warsaw 02-106, Poland; ^4^ Institute for Integrative Biology of the Cell (I2BC), Université Paris-Saclay, CEA, CNRS, Université Paris-Sud, 1 avenue de la terrasse, Gif-sur-Yvette 91198, France; ^5^ Institut National de la Santé et de la Recherche Médicale UMR1078, Université de Bretagne Occidentale, Faculté de Médecine et des Sciences de la Santé, Etablissement Français du Sang (EFS) Bretagne, CHRU Brest, Hôpital Morvan, Laboratoire de Génétique Moléculaire, Brest F-29200, France; ^6^ UMR CNRS 6214-INSERM U1083, Angers 49933, Cedex 9, France; ^7^ Département de Biochimie et Génétique, Centre Hospitalier Universitaire d'Angers, Angers 49933, Cedex 9, France; ^8^ Institute for Integrative Biology of the Cell (I2BC), Université Paris-Saclay, CEA, CNRS, Université Paris-Sud, rue Gregor Mendel, Orsay 91405, France; ^9^ Inserm U1163, Hôpital Necker-Enfants-Malades, Institut Imagine, Université Paris Descartes-Sorbonne Paris Cité, 149 rue de Sèvres, Paris 75015, France; ^10^ Stanford Genome Technology Center, Department of Biochemistry, Stanford University, Palo Alto, CA 94304, USA; ^11^ Department of Genetics, Stanford University School of Medicine, Stanford, CA 94305-5301, USA

**Keywords:** OXPHOS, Drug screening, Genetic suppressors, Mitochondrial disease, Yeast

## Abstract

Mitochondrial diseases are severe and largely untreatable. Owing to the many essential processes carried out by mitochondria and the complex cellular systems that support these processes, these diseases are diverse, pleiotropic, and challenging to study. Much of our current understanding of mitochondrial function and dysfunction comes from studies in the baker's yeast *Saccharomyces cerevisiae.* Because of its good fermenting capacity, *S. cerevisiae* can survive mutations that inactivate oxidative phosphorylation, has the ability to tolerate the complete loss of mitochondrial DNA (a property referred to as ‘petite-positivity’), and is amenable to mitochondrial and nuclear genome manipulation. These attributes make it an excellent model system for studying and resolving the molecular basis of numerous mitochondrial diseases. Here, we review the invaluable insights this model organism has yielded about diseases caused by mitochondrial dysfunction, which ranges from primary defects in oxidative phosphorylation to metabolic disorders, as well as dysfunctions in maintaining the genome or in the dynamics of mitochondria. Owing to the high level of functional conservation between yeast and human mitochondrial genes, several yeast species have been instrumental in revealing the molecular mechanisms of pathogenic human mitochondrial gene mutations. Importantly, such insights have pointed to potential therapeutic targets, as have genetic and chemical screens using yeast.

## Abstract (precis)

**Summary:** In this Review, we discuss the use of budding yeast to understand mitochondrial diseases and help in the search for their treatments.

## Introduction

**Fig. 1.**
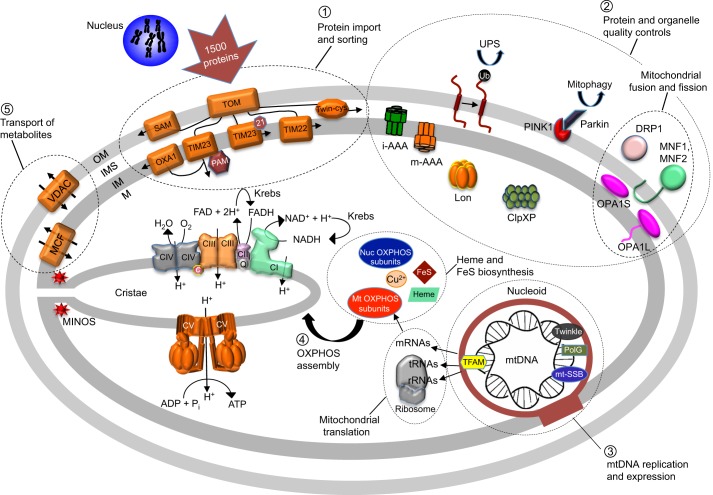
**Overview of mitochondrial processes and components.** This is a schematic cross-section of a human mitochondrion, showing a number of components involved in mitochondrial function. (1) Import and sorting of proteins of nuclear origin: the translocase of the outer membrane (TOM) complex mediates translocation of proteins across or into the outer membrane (OM); sorting and assembly machinery (SAM; also known as TOB) facilitates protein insertion from the intermembrane space (IMS) into the OM; TIM23 takes in proteins with a cleavable mitochondrial targeting sequence (MTS), directing them either into the matrix (M) (when associated to PAM) or the inner membrane (IM) (when associated to Tim21); Twin-cys (the so-called mitochondrial disulfide relay system) mediates, in a redox-dependent manner, the delivery into the IMS of proteins containing specific cysteine motifs; TIM22, together with small soluble proteins in the IMS (called Tim), delivers into the IM the proteins of the so-called mitochondrial carrier family (MCF) that lack a cleavable MTS; OXA1 helps the insertion of proteins from the matrix into the IM. (2) Mitochondrial quality control: misfolded and damaged mitochondrial proteins and organelles are eliminated by proteases and chaperones present in the IM (i-AAA, m-AAA) or the matrix (Lon, ClpXP) by the cytosolic ubiquitin proteasome system (UPS), and by the PINK1 and Parkin proteins at the surface of mitochondria. Fusion (which is mediated by MNF1, MNF2, OPA1L and OPA1S) and fission (mediated by DRP1) of mitochondria contribute also to mitochondrial quality surveillance. (3) mtDNA maintenance and expression: mtDNA is packaged into structures called nucleoids that contain proteins involved in mtDNA maintenance (PolG, Twinkle, mt-SSB), RNA synthesis (TFAM), and the processing of RNAs into messenger (mRNA), transfer (tRNA) and ribosomal (rRNA) RNAs, which are then used to translate the mtDNA-encoded proteins on mitochondrial ribosomes. (4) OXPHOS assembly: the nDNA-encoded subunits of the OXPHOS system (Nuc OXPHOS subunits; CI-V) assemble with their partner subunits of mitochondrial origin (Mt OXPHOS subunits; all except CII, which is entirely encoded by nDNA) together with their redox prosthetic groups (heme and FeS, which are in part synthetized in the mitochondria, and Cu^2+^). CI-IV together with ubiquinone (Q) and cytochrome *c* (*c*) transfer electrons to oxygen from reduced cofactors (NADH, FADH) produced by the Krebs cycle, which is coupled to the pumping of protons out of the matrix. The protons are transported back into the matrix by CV, which is coupled to ATP synthesis from ADP and inorganic phosphate (Pi) (see Fig. 2 for details). (5) Transport of metabolites: systems in the OM (VDAC; also known as porin) and IM [MCF (mitochondrial carrier family)] enable the transport of small solutes and ions into and outside the organelle. Parts of the IM protrude into the matrix, forming the cristae, at the basis of which narrow tubular structures termed ‘cristae junctions’ are maintained by proteins of the mitochondrial inner membrane organizing system (MINOS) complex.

Mitochondria provide energy to the cells by generating adenosine triphosphate (ATP) molecules through the process of oxidative phosphorylation (OXPHOS) in eukaryotes, which involves the oxidation of nutrients (see Box 1) (Saraste, 1999). They also carry out numerous other conserved vital functions, including lipid and steroid synthesis (Horvath and Daum, 2013), and the biosynthesis of iron-sulfur clusters and heme (Lill et al., 2012; Box 2), among many others (Fig. 1) (Galluzzi et al., 2012). Mitochondria are organized as a network of interconnected double-membrane tubules that is continuously remodeled by fusion and fission (Chen and Chan, 2009; Westermann, 2010). The outer and inner membranes (OM and IM, respectively) delineate two aqueous compartments: the intermembrane space (IMS) and matrix. The IM has a boundary domain beneath the OM, and cristae domains that project internally into the matrix (see Fig. 1).

Box 1. **Mitochondrial oxidative phosphorylation (OXPHOS)**

**Fig. 2.**
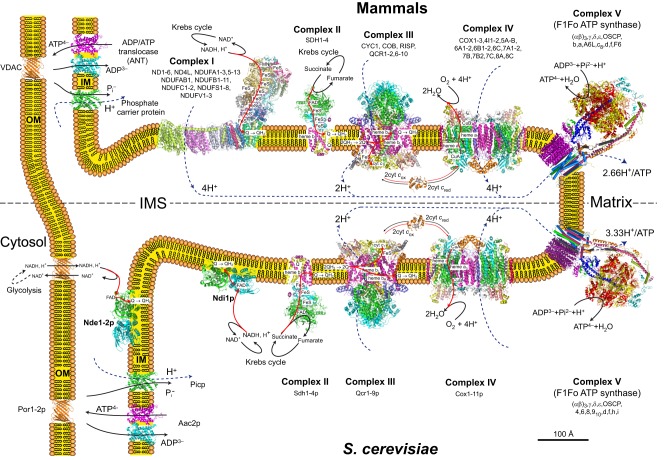
**Mammalian versus yeast OXPHOS system.** The figure shows the main enzymatic systems involved in mitochondrial oxidative phosphorylation (OXPHOS) in yeast and mammals. In mammals (top), complexes I-IV together with ubiquinone (Q) and cytochrome *c* (cyt *c*) transfer electrons to oxygen from the NADH and succinate produced by the Krebs cycle. These transfers are, at the level of complexes I, III and IV, coupled to proton translocation from the matrix into the intermembrane space (IMS). The resulting proton gradient across the inner mitochondrial membrane (IM) is used by complex V (F1Fo ATP synthase) to produce ATP from ADP and inorganic phosphate (Pi). Part of the ATP produced in the matrix is exchanged against cytosolic ADP by the ADP/ATP translocase (ANT) to provide the whole cell with energy and to maintain the ADP phosphorylation capacity of mitochondria. The OXPHOS system of *S. cerevisiae* (bottom) is highly similar to the mammalian system except that complex I is replaced by a non-proton-translocating NADH dehydrogenase (Ndi1p) at the inner side of the IM. There are also in S. *cerevisiae* two NADH dehydrogenases on the external side of the IM (Nde1p, Nde2p) that deliver electrons at the level of ubiquinone. The protein structures are from the Protein Data Bank (PDB) and are at the same scale (indicated by the scale bar).

As a first step in oxidative phosphorylation, fuel molecules (such as monosaccharides and fatty acids) are transferred to nicotinamide (NAD) and flavine adenine (FAD) nucleotides, through glycolysis, the Krebs cycle and β-oxidation (see Box 2 for a glossary of terms), and are then moved to oxygen through an electron transport chain (ETC) (Box 2) typically made of four protein complexes (CI-CIV) embedded within the mitochondrial inner membrane (IM). The free energy released by the electron flow at the level of CI, CIII and CIV is used to maintain an electrochemical potential (ΔµH) (Box 2), composed of an electrical gradient (ΔΨ) and a pH gradient, across the mitochondrial IM. This membrane potential drives ATP synthesis from ADP and inorganic phosphate (Pi) through the activity of an enzyme called F_1_F_O_-ATP synthase (CV) (Fig. 2). Most of the ATP produced by CV is exchanged against cytosolic ADP through a specific adenine nucleotide carrier (ANC), to supply the rest of the cell with energy and to maintain the ADP phosphorylation capacity of mitochondria (Fig. 2). ETC form large and stable supra-molecular structures, called respirasomes (Boekema and Braun, 2007; Dudkina et al., 2011; Winge, 2012; Wittig and Schagger, 2009), which enhance substrate channeling and reduce the production of reactive oxygen species (ROS; see Box 2) caused by the diversion of electrons from their normal pathway to oxygen. CV also forms oligomeric structures in the form of ribbons of dimeric units that are important for the formation of mitochondrial cristae (Fig. 1) (Giraud et al., 2002; Habersetzer et al., 2013; Paumard et al., 2002; Strauss et al., 2008).

Box 2. **Glossary of terms**

**Aminoaciduria:** presence of amino acids in the urine that can be increased by metabolic disorders, chronic liver disease or renal disorders.

**Aminoacyl tRNA synthetase:** an enzyme attaching the appropriate amino acid onto its tRNA, an essential step in the synthesis of proteins.

**Autophagy:** a mechanism enabling the cell to degrade and recycle unnecessary or dysfunctional components.

**β-oxidation:** the process that transforms fatty acids into acetyl-CoA, which is then oxidized by the Krebs cycle.

**Cholestasis:** a condition in which bile cannot flow from the liver to the duodenum.

**Citrate:** conjugate base of citric acid, which is an important intermediate in the citric acid (or Krebs) cycle.

**Corpus callosum:** a wide, flat bundle of neural fibers beneath the cortex that connects the left and right cerebral hemispheres.

**Cybrid (cytoplasmic hybrid):** a eukaryotic cell line produced by the fusion of a whole cell lacking mitochondrial (mt)DNA (ρ^0^) with an enucleated cell (cytoplast), which can be used to investigate the pathogenesis of mtDNA in individuals with a mitochondrial disease in a control nuclear genetic background.

**Cyclic neutropenia:** a disorder causing frequent infections due to a shortage of neutrophils, which are a type of white blood cell that play a role in inflammation and in fighting pathogens such as bacteria and viruses.

**Cytochrome *c*:** a soluble hemoprotein in the intermembrane space of mitochondria that transfers electrons from CIII to CIV.

**Electrochemical potential (ΔµH):** a gradient of electrical potential and chemical concentration enabling the movement of ions (e.g. protons) across a biological membrane.

**Electron transport chain (ETC):** a multicomponent system – usually a series of multi-subunit protein complexes – that transfers electrons from one molecule to another.

**Flavine adenine dinucleotide (FAD):** a redox cofactor involved in the transfer of electrons from one molecule to another.

**Glycolysis:** a metabolic pathway that converts glucose into pyruvate.

**Heme *a*_3_:** a coordination complex attached to CIV that consists of an iron atom chelated by porphyrin and which can bind dioxygen.

**Heteroplasmy:** a term used in genetics to describe mammalian cells whose copies of mtDNA are not all identical, which is an important factor in considering the severity of diseases caused by mutations in the mitochondrial genome. Mitochondrial heteroplasmy in disease is the co-existence within a cell of wild-type and mutated mitochondrial (mt)DNA; beyond a certain threshold mutated mtDNA results in deleterious physiological consequences.

**Homoplasmy:** a term used in genetics to describe a mammalian cell whose copies of mtDNA are all identical, either normal or mutated.

**Hyperornithinemia-hyperammonemia-homocitrullinuria (HHH):** a metabolic disorder that can result in chronic neurocognitive deficits (including developmental delay, ataxia, spasticity, learning disabilities and seizures), acute encephalopathy and chronic liver dysfunction.

**Intergenic region:** a stretch of DNA located between genes.

**Intron:** any nucleotide sequence within a gene that is removed by RNA splicing.

**Iron-sulfur (Fe-S) proteins:** proteins characterized by the presence of iron-sulfur clusters that are used in oxidation-reduction reactions, such as proteins belonging to complexes I, II and III of the mitochondrial electron transport chain (ETC).

**Krebs cycle:** also known as the citric acid cycle or the tricarboxylic cycle (TCA); a series of chemical reactions enabling aerobic organisms to generate energy through the oxidation of acetate derived from carbohydrates.

**Leigh syndrome:** a rare inherited neurometabolic disorder affecting the central nervous system.

**Lon:** ATP-dependent protease (also called protease La) whose name is derived from the phenotype of *Escherichia coli lon* gene mutants that form long (hence the name Lon) undivided filaments upon UV radiation.

**Mitophagy:** the process by which mitochondria are degraded via the autophagy pathway.

**Neuropathy, ataxia and retinitis pigmentosa (NARP):** a rare disease with maternal inheritance that chiefly affects the nervous system and is characterized by various symptoms such as pain in the arms and legs, muscle weakness, loss of vision, and problems with balance and coordination.

**Nicotinamide adenine nucleotide (NAD):** a redox cofactor found in all living cells that is involved in the transfer of electrons from one molecule to another.

**Ornithine:** an amino acid that plays a role in the urea cycle, which allows the disposal of excess nitrogen.

**Reactive oxygen species (ROS):** chemically reactive oxygen-containing molecules that can damage any type of biomolecule.

**Ubiquinone (coenzyme Q):** a hydrophobic component with high mobility in biological membranes that transfers electrons from CI and CII to CIII in mitochondria.

Mitochondrial genomes are remnants of an ancestral prokaryotic genome, most of which has been lost or transferred to the nucleus during the evolution of eukaryotes (Gray, 2014; Gray et al., 1999). Thus, most of the genes required for mitochondrial structure and function (>99%) are located in the nucleus of the cell [the nuclear DNA (nDNA)] and a tiny proportion is located in mitochondria (mtDNA). nDNA-encoded mitochondrial proteins are synthesized by cytoplasmic ribosomes and imported into mitochondria (Dolezal et al., 2006; Harbauer et al., 2014; Neupert and Herrmann, 2007). The maintenance of a separate genetic system in mitochondria is costly because it requires numerous proteins for: mtDNA replication, repair, recombination and transcription; mitochondrial RNA (mtRNA) processing and translation; and for gene regulation in the organelle (Fox, 2012; Herrmann et al., 2013; Peralta et al., 2012). Moreover, nuclear-mitochondrial interactions need fine-tuning to coordinate the expression of both nuclear and mitochondrial genomes (Frechin et al., 2014).

Given the structural and functional complexity of mitochondria, it is not surprising that mitochondrial dysfunction has been implicated in a broad spectrum of human diseases. The first case was reported in 1959 by Roth Luft, who described a young woman suffering from a hypermetabolic disorder in the form of excessive mitochondrial respiration not effectively coupled to ATP production (Ernster et al., 1959). Since then, more than 150 distinct genetic mitochondrial dysfunction syndromes have been described, most of which arise from disorders affecting the energetic function of mitochondria. These diseases affect at least 1 in 5000 live human births (Skladal et al., 2003) and can present either in infancy or adulthood, in a multisystemic or highly tissue-specific manner. Typical clinical traits include visual and/or hearing defects, encephalopathies, cardiomyopathies, myopathies, diabetes, and liver and renal dysfunctions (DiMauro and Schon, 2003; Vafai and Mootha, 2012; Zeviani and Carelli, 2007). Many known cases result from alterations in mtDNA, which occur as a result of this DNA's high susceptibility to mutations because of the nearby production of reactive oxygen species (ROS; Box 2) and the poor effectiveness of the mtDNA repair system (Wallace, 2010). Mitochondria are also believed to have a role in common disorders, such as diabetes, obesity, age-related neurodegenerative and cardiovascular diseases, cancer, and probably also the aging process owing to a progressive decline of mitochondrial function during life (Koopman et al., 2013; Wallace, 2012).

Despite considerable progress in defining the pathogenesis of mitochondrial disorders over the last 20 years, there are still no effective therapies to treat them. Although gene-therapy approaches have been envisioned for treating some mitochondrial diseases (DiMauro et al., 2006; Schon et al., 2010), a strategy that would be easier to implement is effective metabolic or pharmacological treatment (Andreux et al., 2013; Wallace et al., 2010). Many pharmacological agents have been assessed as therapies for mitochondrial diseases, including vitamins or cofactors involved in energy metabolism, metabolic intermediates, enzyme activators and anti-oxidants, but none has so far shown conclusive therapeutic benefit (Kerr, 2010, 2013). Thus, there is an urgent need to develop new drugs to treat these diseases.

Recent work, reviewed here, has proven that yeast is a valuable system in which to model mitochondrial disorders and identify new compounds with therapeutic potential. As the organism in which mtDNA was first discovered (Corneo et al., 1966; Ephrussi and Slonimski, 1955; Mounolou et al., 1966), *Saccharomyces cerevisiae* has played an essential role in building our understanding of mitochondrial function. This includes providing details of mitochondrial regulation, its key processes and components, and their interplay with cellular functions. Not only has this understanding been important for studies of these processes in humans and other eukaryotes, but the methodologies developed in yeast have also enabled detailed investigations of pathogenic mutations implicated in mitochondrial diseases. The success of such studies with this unicellular and relatively simple eukaryote hinges on several factors: the high conservation of mitochondrial function, the exceptionally thorough annotation of the yeast genome, and the tractability of yeast for manipulations of the mitochondrial and nuclear genomes and for genetic and chemical screens.

## Mitochondrial structure and function

We begin this Review by providing a brief overview of mitochondrial processes and components that have been implicated in human diseases.

### The OXPHOS system

The energy-transducing system of mitochondria comprises multi-subunit complexes (CI-CV) embedded within the mitochondrial IM, which, together with cytochrome *c* and ubiquinone (see Box 2 for a glossary of terms), form what is usually called the OXPHOS system (see Box 1, and Figs 1, 2). CI-IV transfer electrons to oxygen, a process that is coupled to the pumping of protons out of the mitochondrial matrix; protons are transported back into the matrix by CV (ATP synthase), which is coupled to ATP synthesis from ADP and inorganic phosphate. The OXPHOS system contains approximately 90 different structural protein subunits [of which 13 are encoded by the mtDNA in humans (Box 3)] and 24 redox groups [13 FeS, one flavin mononucleotide (FMN), one flavine adenine (FAD), seven hemes and two Cu^2+^; see Box 2 and Fig. 2]. Assembling this system is a sophisticated process involving dozens of proteins with highly specific actions (Devenish et al., 2008; Fox, 2012; Ghezzi and Zeviani, 2012; Mick et al., 2011; Nouws et al., 2012; Rak et al., 2009; Rutter et al., 2010; Smith et al., 2012; Vogel et al., 2007; Zara et al., 2009).

Box 3. **The human mtDNA genome**

The human mitochondrial genome is a compact, double-stranded, circular DNA molecule of 16,569 bp that encodes 13 energy-transducing proteins [seven CI subunits (ND1, ND2, ND3, ND4, ND4L, ND5 and ND6), one CIII subunit (cytochrome *b*), three CIV subunits (COX1, COX2, COX3) and two CV subunits (ATP6, ATP8)], and 22 tRNAs and two rRNAs that are required for protein synthesis inside the organelle (Andrews et al., 1999). The human mtDNA contains no introns and almost no intergenic sequences (see Box 2 for a glossary of terms), with the exception of the 1.1-kb displacement loop (D-loop) where transcriptional promoters and at least one of the proposed replication origins (O_H_) are located. Core components of the human mtDNA replication machinery include the mitochondrial γ polymerase (POLG), consisting of a catalytic subunit with 5′-3′ exonuclease activity (PolgA) and a processivity subunit (PolgB), a protein with 5′-3′ DNA helicase activity (Twinkle) and single-stranded binding protein (mt-SSB) (Holt and Reyes, 2012; Rotig and Poulton, 2009). Most of the mtDNA genes are transcribed as almost genome-length polycistronic transcripts (see Box 2) that are next processed to produce individual mRNA and tRNA molecules. Core components of the mitochondrial transcription machinery include RNA polymerase (POLRMT), the transcription activator A (TFAM), the transcription factor TFB2M (transcription factor B2, mitochondrial) and the termination factor mTERF (mitochondrial transcription termination factor) (Bestwick and Shadel, 2013). Excision of tRNAs from polycistronic transcripts involves two RNases, P and Z (Holzmann et al., 2008; Takaku et al., 2003). Most mRNAs are polyadenylated by MTPAP [mitochondrial poly(A) polymerase] (Tomecki et al., 2004), which is believed to regulate their stability and is often required to generate their stop codon (Nagaike et al., 2005; Wydro et al., 2010). Proteins involved in mitochondrial protein synthesis include: ribosomal proteins, aminoacyl tRNA synthetases, mt-tRNA modification enzymes, two initiation factors (IF2 and IF3), three elongation factors (EFG1, EFTs and EFTu), at least one termination release factor (mtRF1), the translation regulator PTCD3 (pentatricopeptide repeat domain 3), the ribosome recycling factors mtRFF and EFG2, and the methionine aminopeptidase MAP1D, which removes N-terminal methionine (Christian and Spremulli, 2012; Lightowlers et al., 2014).

### Mitochondrial protein import

After their synthesis in the cytosol, nDNA-encoded mitochondrial proteins must be imported and sorted to their respective intra-mitochondrial locations: the outer membrane (OM), the intermembrane space (IMS), the inner membrane (IM) or the matrix. This process is mediated by a multi-component machinery [reviewed in Chacinska et al., 2009; Dolezal et al., 2006; Fox, 2012; Harbauer et al., 2014; see also Fig. 1 and its legend], the activity of which is modulated at multiple levels to regulate biogenesis, composition and turnover of the organelle in connection with cellular metabolism, signaling and stress (Harbauer et al., 2014).

### mtDNA maintenance and expression

The human mtDNA genome is inherited through the maternal lineage (Giles et al., 1980). It is packaged into structures termed nucleoids (Bogenhagen, 2012; Kukat and Larsson, 2013) – which frequently contain a single copy of mtDNA (Kukat et al., 2011) – that contain proteins involved in mtDNA replication, maintenance, repair and recombination (Box 3, Fig. 1). Dozens of nuclear-encoded proteins are additionally required for mitochondrial protein synthesis. Because all the intraorganellar-synthesized proteins are very hydrophobic, they can have different chaperone requirements than soluble proteins synthesized on cytosolic ribosomes. Specific subunits of the mitochondrial ribosome (MRPL39, MRPL44 and MRPL45) have evolved to facilitate the accurate co-translational insertion of the nascent peptide into the IM together with the help of various factors (Greber et al., 2014). In yeast mitochondria, messenger RNA (mRNA) recognition by the ribosome utilizes the untranslated region upstream of the start codon (5′UTR) to establish interactions with gene-specific IM-located translational activators (Fox, 2012). Mammalian mitochondrial mRNAs do not have 5′UTRs; as-yet-unknown alternative mechanisms must therefore be responsible for their recognition by the ribosome. So far, only a limited number of factors involved in mitochondrial ribosome biogenesis have been identified (Dennerlein et al., 2010; Kehrein et al., 2015; Metodiev et al., 2009; Tu and Barrientos, 2015). An interesting recent development indicates that the mitochondrial ribosomal RNAs (mt-rRNAs) assemble with mitochondrial ribosomal proteins immediately after, or concomitant with, their synthesis (Bogenhagen et al., 2014; Dalla Rosa et al., 2014).

### Protein and organelle quality controls

Co-ordinating the expression of nDNA and mtDNA is of crucial importance to avoid the accumulation of unassembled OXPHOS subunits, which are prone to misfolding or aggregation. Mitochondria also have to deal with the production of deleterious ROS, created by the OXPHOS system, which damage biomolecules and ultimately lead to the depolarization of the IM and to apoptosis (Martinou and Youle, 2011; Wang and Youle, 2009). Mitochondria have therefore evolved quality-control mechanisms to remove damaged proteins and to sequester and eliminate damaged organelles (Anand et al., 2013; Baker and Haynes, 2011; Baker et al., 2011) (see Fig. 1). A cohort of mitochondrial proteases enables the clearing of damaged proteins from the IM [m-AAA [AAA (ATPase Associated with diverse Activities) protease that is active at the matrix side of the mitochondrial membrane), i-AAA (protrudes into the IMS) and OMA1 (has overlapping activity with the m-AAA protease)] and the matrix [Lon (see Box 2) and ClpXP]. Nuclear-encoded mitochondrial proteins that become mistargeted or misfolded *en route* to the organelle are removed by the cytosolic ubiquitin-proteasome system (UPS) (Livnat-Levanon and Glickman, 2011), which can also degrade proteins residing in the OM (Karbowski and Youle, 2011). Mitochondria also undergo fusion and fission events to enable them to maintain their shape, number, functional properties and integrity of their genome (Osman et al., 2015). This activity is mediated by evolutionary conserved GTPases of the dynamin superfamily located in the mitochondrial membranes [dynamin-related protein 1 (DRP1), mitofusin 1/2 (MFN1/2) and optic atrophy 1 (OPA1); see Fig. 2]. Fusion allows mitochondria that are deficient in some components to be replenished with healthy organelles. Under certain stress conditions, the mitochondrial network becomes hyper-fused, which protects mitochondria against autophagy (see Box 2) and maintains cellular ATP production (Rambold et al., 2011). Severely damaged mitochondria that are unable to sufficiently energize the IM can no longer fuse, which results in their separation from the mitochondrial network and subsequent degradation by autophagy, a phenomenon called mitophagy (see Box 2) (Kim et al., 2007; Sauvanet et al., 2012; Twig et al., 2008). A central role for the autophagic removal of depolarized mitochondria has been assigned to the E3 ubiquitin ligase Parkin and PTEN-induced kinase 1 (PINK1): functional mitochondria efficiently import and degrade PINK1; when the IM potential collapses, PINK1 accumulates at the mitochondrial surface, where it recruits Parkin to initiate mitophagy (Matsuda et al., 2010; Narendra et al., 2010). Previously unknown lines of defense against mitochondrial damage are emerging, like the delivery of selective mitochondrial cargo to lysosomes as an early response to oxidative stress (Soubannier et al., 2012).

In addition to their roles in degrading misfolded or oxidatively damaged proteins, mitochondrial proteases have key regulatory functions. For example, the Lon protease prevents excessive accumulation of the mitochondrial transcription factor TFAM. Excessive accumulation of TFAM relative to mtDNA copy number inhibits transcription (Matsushima et al., 2010). The Lon protease additionally affects the biosynthesis of steroid hormones by degrading a protein (StAR) that facilitates cholesterol transfer from the outer to the inner mitochondrial membranes, making it available for steroid hormone synthesis (Lin et al., 1995). A key regulatory function of m-AAA protease is the processing of the precursor form of the Mrp32 subunit of the mitochondrial ribosome, a prerequisite for its assembly and hence for mitochondrial translation (Bonn et al., 2011). In addition to its clearance activity of damaged proteins, the i-AAA protease has a central role in the maintenance of the lipid composition of mitochondrial membranes (Nebauer et al., 2007; Potting et al., 2010).

### Transport of metabolites

Because the IM is largely impermeable to solutes and ions, proper mitochondrial function requires a cohort of systems within this membrane for the import into the matrix of numerous small molecules, such as ADP and inorganic phosphate for oxidative phosphorylation and substrates of the Krebs cycle (Box 2), as well as for the export into the cytosol of a number of molecules produced inside the organelle, such as ATP and heme-biosynthesis intermediates. Most of this transport is mediated by structurally related proteins referred to as mitochondrial carriers (MCs) (Palmieri, 2014).

## Yeast as a model for studying human mitochondrial diseases

Much of what we know about mitochondria originally came from studies in *S. cerevisiae*, which was established as a genetic system for studying mitochondria 60 years ago, by Boris Ephrussi and Piotr Slonimski, owing to its ability to survive mutations that inactivate OXPHOS when provided with fermentable sugars (Ephrussi and Slonimski, 1955). The first characterized respiratory-deficient *S. cerevisiae* mutation (ρ^−^/ρ^0^) revealed that respiration is controlled in yeast by a non-mendelian genetic element, the ρ factor, which was found a couple of years later to be a small DNA molecule located in the mitochondrion (mtDNA) (Corneo et al., 1966; Mounolou et al., 1966). Because of the limited coding capacity of mtDNA, it was rapidly realized that most of the fundamental processes involved in mitochondrial biogenesis must depend on the expression of genes located in nDNA. This was confirmed by Alexander Tzagoloff, who established more than 30 years ago the existence of at least 200 genetically distinct nuclear loci required for the growth of yeast cells on non-fermentable substrates, many of which were shown to control the replication and expression of mitochondrial genes (Tzagoloff and Dieckmann, 1990). The sequencing of the *S. cerevisiae* genome and the construction of a whole-genome deletion-mutant collection identified 265 previously unknown nuclear genes required for optimal respiratory growth (Steinmetz et al., 2002). Proteomic analyses have also been used to explore the protein composition of this organelle. Mass spectrometry analyses of highly pure yeast mitochondria identified 850 proteins and led to an estimation that there are in total about 1000 protein species in yeast mitochondria (Prokisch et al., 2004; Reinders et al., 2006). Remarkably, a similar number of mitochondrial proteins was estimated from the analysis of 14 different mouse tissues, of which more than 50% had a yeast homolog (Pagliarini et al., 2008), which indicated that the mitochondria of single-celled organisms are as complex, and are highly similar, to those found in individual tissues of higher eukaryotes. Further analyses have revealed that the mammalian mitochondrial proteome likely contains 1500 proteins, of which 1100 have been identified (Rhee et al., 2013). Tissue diversity is a likely reason for the larger size of the mitochondrial proteome in mammals compared to yeast. As described in the next section, the high similarities between yeast and human mitochondria considerably helped the study of mitochondrial diseases.

### Yeast models of human diseases caused by mtDNA point mutations

Currently, more than 250 point mutations in human mtDNA that are proven or suspected to be pathogenic have been identified (http://www.mitomap.org). Mutations in protein-encoding mitochondrial genes primarily (and possibly only) affect the energy-transducing complexes to which they belong, whereas mutations in mitochondrial transfer RNA (mt-tRNA) genes have more pleiotropic consequences by impairing mitochondrial protein synthesis. mtDNA point mutations are often heteroplasmic (see Box 2), and are usually considered as being highly recessive (relative to the corresponding wild-type alleles), which can render it difficult to evaluate how they affect mitochondrial functions. Furthermore, given the high mutational rate of the mitochondrial genome and the presence of numerous family or population-specific polymorphisms, it can be difficult to distinguish between a neutral mtDNA variant and a disease-causing mutation. Also, multiple studies have determined that the effects of deleterious mtDNA mutations might be exacerbated by mtDNA nucleotide changes that are not pathogenic per se and by unknown factors in nuclear genetic background, i.e. so-called modifier genes (Cai et al., 2008; Swalwell et al., 2008).

**Fig. 3.**
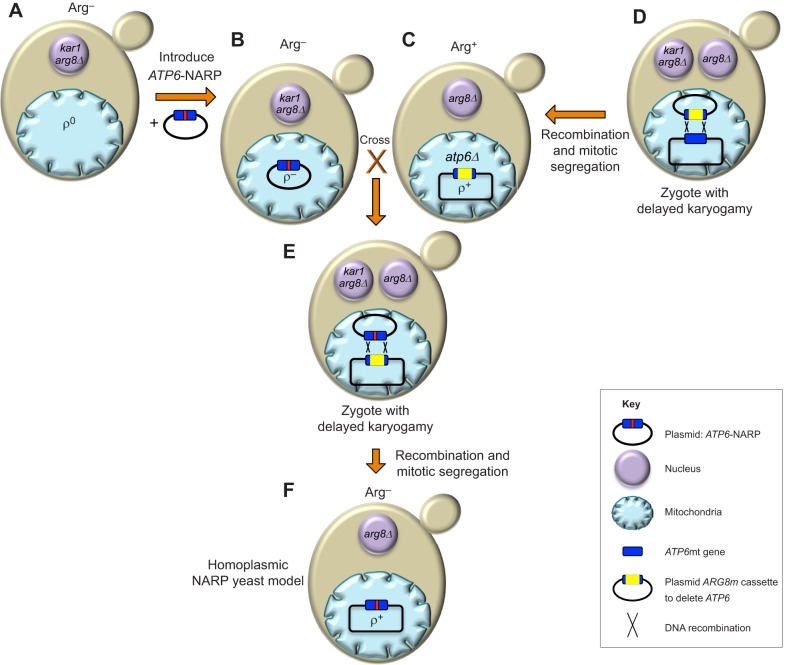
**Construction of yeast models of a human mtDNA pathogenic mutation.** Schematic of the steps used to create a yeast model of a mutation of the human mitochondrial *ATP6* gene, which causes neuropathy ataxia retinitis pigmentosa (NARP) syndrome. (A) In this approach, a plasmid containing a mutant version of the yeast *ATP6* gene that carries the NARP-associated mutation is created (*ATP6*-NARP). This is introduced into the mitochondria of a ρ^0^ *arg8Δ kar1* strain, which is devoid of mtDNA (ρ^0^), has a null allele of the nuclear *ARG8* gene (*arg8Δ*) and a mutation (*kar1*) that strongly delays nuclear fusion, which allows the transfer of mtDNA from one nuclear haploid background to another (Conde and Fink, 1976). (B) The resulting ρ^−^ synthetic strain, which fails to grow in the absence of external arginine (ARG^−^), is crossed with (C) an arginine prototrophic (ARG^+^) strain that contains wild-type (ρ^+^) mtDNA but is deleted for *ATP6* (*atp6Δ*). *ARG8m* is a mitochondrial version of a nuclear gene (*ARG8*) and encodes a yeast mitochondrial protein involved in arginine biosynthesis (Steele et al., 1996). (D) Because the *ARG8m* clone used to delete *ATP6* is flanked on each side by ∼100 bp of the *ATP6* locus, homologous recombination (E) can mediate the replacement of *ARG8m* with the *ATP6*-NARP gene. (F) Mitotic segregation then produces ρ^+^ cells with the NARP-associated *ATP6* mutation in a pure (homoplasmic) form that can be identified by virtue of their inability to grow in the absence of arginine.

Owing to the absence of methods to mutagenize the mitochondrial genomes of mammals, *S. cerevisiae* has been utilized as an alternative model to investigate mtDNA mutations found in patients. Mitochondrial genetic transformation can be achieved in *S. cerevisiae* in a highly controlled fashion, by the biolistic delivery (transfection by bombardment with DNA-coated gold particles using a ‘gene gun’) into mitochondria of *in-vitro*-made mutated mtDNA fragments, followed by their integration into wild-type mtDNA by homologous DNA recombination (Bonnefoy and Fox, 2001) (Fig. 3). Being unable to stably maintain heteroplasmic mtDNA (Okamoto et al., 1998), it is relatively easy to obtain yeast homoplasmic populations in which all mtDNA molecules carry a mutation of interest. Several groups have exploited these attributes to study various pathogenic mtDNA mutations – for example, in the genes encoding subunits of complexes III (cytochrome *b*) and IV (COXI, COXIII) (Meunier, 2001; Meunier et al., 2013), and in mt-tRNA genes (Feuermann et al., 2003; Montanari et al., 2008), which have helped to better define the functional consequences of these mutations. We similarly investigated seven mutations (T9176G, T8851C, T8993G, T9191C, T9176C, T8993C and T9185C) of the mitochondrial *ATP6* gene found in individuals with neuropathy, ataxia and retinitis pigmentosa (NARP; see Box 2), Leigh syndrome (LS; see Box 2) or bilateral striatal lesions of childhood (BSLC) (Kabala et al., 2014; Kucharczyk et al., 2010, 2013, 2009a,b; Rak et al., 2007). All these mutations significantly decrease the rate of mitochondrial ATP synthesis in yeast, by 30 to >95% compared with the wild type. Our study of T8851C has confirmed its previously uncertain pathogenicity by revealing a block in the proton-translocating domain of ATP synthase (Kucharczyk et al., 2013). Although only a few cases of this mutation have been reported in patients, these results support that it is responsible for the BSLC disorder and premature death of affected individuals. We have also shown that the T9176G mutation severely impedes the incorporation of the protein encoded by the *ATP6* gene (which is referred to as subunit *a* or subunit 6) into yeast ATP synthase (Kucharczyk et al., 2009b), and evidence for similar defects has been reported in skin fibroblasts from patients carrying this mutation (Carrozzo et al., 2001). Importantly, these findings in yeast correspond to the reported severity of these mutations in humans, which likely reflects a high level of evolutionary conservation within the regions of subunit *a*/6 that they affect (Baracca et al., 2000, 2007; Carrozzo et al., 2000, 2004; Cortes-Hernandez et al., 2007; De Meirleir et al., 1995; Dionisi-Vici et al., 1998; Houstek et al., 2006; Mattiazzi et al., 2004; Morava et al., 2006).

### Yeast and nDNA-based mitochondrial diseases

The first nuclear mutation responsible for a mitochondrial disease was discovered in 1995, in the subunit A of CII (SDHA), in two siblings affected with LS (Bourgeron et al., 1995). Since then, over 150 nuclear genes involved in mitochondrial diseases have been identified (Calvo et al., 2010; Koopman et al., 2012; Vafai and Mootha, 2012), 70% of which are conserved in yeast (see supplementary material Table S1). As we discuss below, the similarities between yeast and human mitochondria, and the experimental benefits of the yeast system, have helped to resolve the genetic and biochemical underpinnings of numerous mitochondrial diseases with a nuclear genetic origin.

#### Diseases caused by OXPHOS assembly defects

After the discovery of the yeast *ATP12* gene and its role in the assembly of the catalytic head of CV (Ackerman and Tzagoloff, 1990), sequence comparisons identified a similar gene in human cDNA databases (called *ATPAF2*) that proved to function like its yeast counterpart, as tested by heterologous complementation (Wang et al., 2001). A mutation in *ATPAF2* was subsequently found as being responsible for the death of a 14-month-old girl who had severe neurological defects due to a low CV content (De Meirleir et al., 2004).

Similarly, after the discovery of the yeast proteins Bcs1p [full name: ubiquinol-cytochrome *c* reductase (*bc*_1_) synthesis] (Cruciat et al., 1999) and Mzm1p (mitochondrial zinc maintenance) (Cui et al., 2012) as being required for the maturation and/or insertion of the Rieske iron-sulfur protein (Rip1) into CIII, the homologous human genes, *BCS1L* and *MZM1L*, were considered as obvious candidates in individuals with nDNA-based CIII-assembly defects. Indeed, *BCS1L* (de Lonlay et al., 2001) and *MZM1L* (Invernizzi et al., 2013) mutations were found in such individuals from unrelated families, and complementation assays in yeast supported the deleterious nature of several of them (Ostojic et al., 2013).

Another example of a protein involved in OXPHOS assembly is Sdh5p, a conserved protein of unknown function identified in the yeast mitochondrial proteome (Sickmann et al., 2003); this protein is called SDHAF2 (succinate dehydrogenase assembly factor 2) in humans. It proved to be necessary and sufficient for the attachment of FAD (see Box 2) in CII (Hao et al., 2009), and the human homolog was subsequently shown to interact with CII and was able to rescue the flavination of the yeast enzyme in an *sdh5Δ* deletion mutant, suggesting functional conservation. Based on the causal relationship of loss of CII activity with a rare neuroendocrine tumor called paraganglioma (PGL), the human *SDHAF2* gene was screened for mutations in such affected individuals. One indeed had a mutation in this gene and the mutated gene was unable to complement yeast *sdh5Δ* strains, strongly suggesting that the mutation was causative.

Yeast has also helped to resolve the most common cause of LS, which is associated with a CIV deficiency linked to a region of chromosome 9q. One gene in this region, *SURF-1*, which belongs to the so-called surfeit cluster of six very tightly linked genes that do not share sequence similarity, was found to display homology with a previously identified yeast gene (called *SHY1*, for *SURF-1* homolog in yeast) that encodes a mitochondrial protein required for CIV expression and respiration (Mashkevich et al., 1997). Dozens of individuals from unrelated families with a CIV deficiency were subsequently shown to carry mutations in *SURF-1*, and further studies determined that the proteins encoded by *SHY1* and *SURF-1* are both required for inserting heme *a*_3_ (see Box 2) into CIV (Mashkevich et al., 1997; Pecina et al., 2004). Similarly, mutations causing distinct clinical phenotypes – cardioencephalopathies and hepatopathies – were found in two genes, *SCO1* and *SCO2*, respectively, that are homologous to a yeast gene (*SCO1*) required for copper delivery into CIV (Leary et al., 2004; Papadopoulou et al., 1999; Valnot et al., 2000a). In line with these findings, copper supplementation was found to restore CIV activity in patient cells carrying mutations in *SCO2* (Casarin et al., 2012). Recently, a homozygous mutation in individuals from two unrelated families displaying ataxia and muscle hypotonia was found in a gene (*FAM36A*) homologous to the yeast gene *COX20* (Szklarczyk et al., 2013), which has previously been shown to encode a protein that assists membrane insertion and maturation of the COXII subunit of CIV (Elliott et al., 2012). It was thus expected that these individuals had defects in the assembly of CIV, which was confirmed (Bourens et al., 2014; Szklarczyk et al., 2013). Another well-illustrative example of CIV-based disease that yeast helped to resolve involves mutations in the gene *COX10*, which encodes a protein with farnesyl transferase activity, which is required for heme *a* maturation (Tzagoloff et al., 1993; Valnot et al., 2000b).

The absence of CI in *S. cerevisiae* has been exploited in the search for assembly factors of this complex using a comparative genomics approach with CI-containing yeast species, such as *Yarrowia lipolytica*. These studies identified the protein B17.2 and then its human homolog (B17.2L), which proved to be an essential chaperone of CI and in which mutations were found in an individual displaying progressive encephalopathy (Ogilvie et al., 2005). Similarly, another evolutionarily conserved protein involved in CI assembly [Ind1 in *Y. lipolytica*; NUBPL (nucleotide-binding-like) in humans] that is required for inserting Fe-S centers (see Box 2) in the peripheral arm of this complex was found, and mutations of this protein were associated with encephalopathies (Calvo et al., 2010; Sheftel et al., 2009). These findings from yeast provide new molecular insights into OXPHOS assembly defects, and into the complex regulation of this system's biogenesis, and will likely reveal more insights in the future.

#### Diseases characterized by mtDNA maintenance defects

More than 200 mutations in the gene *POLG* (polymerase gamma), which encodes the catalytic component of mtDNA polymerase, have been implicated in various diseases, including progressive external ophtalmoplegia (PEO), Alper's syndrome (also called Alper-Huttenlocher syndrome), myopathy, parkinsonism (a neurological syndrome characterized by tremor, rigidity and postural instability that shares symptoms found in Parkinson's disease), premature menopause, psychological disorders and ataxia-neuropathy syndrome (http://tools.niehs.nih.gov/polg) (Hudson and Chinnery, 2006; Stumpf and Copeland, 2011). These diseases result from depletion, large-scale deletions and/or point mutations in mtDNA that compromise mitochondrial function. Owing to its ability to survive mtDNA loss, a property referred to as ‘petite-positivity’ shared by only a few yeast species (Bulder, 1964; Chen and Clark-Walker, 2000), *S. cerevisiae* is an ideal system in which to investigate the functional consequences of *POLG* mutations. In particular, yeast have helped to distinguish between truly pathogenic and harmless single-nucleotide polymorphisms (SNPs), and to determine whether deleterious mutations are dominant or recessive, and whether they impact POLG stability or locally disturb domains that are important for the processing and fidelity of mtDNA replication. For instance, studies in yeast revealed that the T654A and R656Q *POLG* mutations are dominant, slow down replication and result in higher mtDNA mutability (Baruffini et al., 2006). Mutations affecting the exonuclease domain of POLG, which is responsible for the fidelity of mtDNA replication, are generally less detrimental, causing only modest increases in the rate of mtDNA mutation (Szczepanowska and Foury, 2010). As with the yeast *ATP6* models of diseases (see above), pathogenic *POLG* mutations produce a similar degree of phenotypic severity in both yeast and humans.

Mutations in a small protein of yet-unknown function encoded by the gene *MPV17* was determined as a main cause of mitochondrial DNA depletion syndrome (MDS), which predominently affects the liver and eventually induces neurological degeneration (Spinazzola et al., 2006). Its yeast homolog, *SYM1*, is required for ethanol tolerance and for maintaining the mitochondrial morphology under heat stress (Trott and Morano, 2004). The human *MPV17* gene can complement *sym1Δ* deletion strains, indicating functional conservation (Trott and Morano, 2004). Although the loss of *SYM1* leads to a higher production of ρ^−^/ρ^0^ petites lacking functional mtDNA, this effect is rather mild, indicating that *MPV17*-based diseases possibly have an origin other than a failure in mtDNA propagation (Dallabona et al., 2010).

#### Diseases caused by defects in mitochondrial protein import

Because most mitochondrial proteins are encoded by nDNA, defects in the mitochondrial protein import process can have widespread effects. Mutations affecting GFER (full name: growth factor homolog to yeast ERV1 responsible for liver regeneration in humans) (Di Fonzo et al., 2009) and DPP1 (deafness dystonia peptide 1) (Aguirre et al., 2006; Jin et al., 1996), two components of the mitochondrial protein import machinery, were found in individuals presenting with multiple mitochondrial deficiencies and a complex clinical phenotype characterized by visual and/or hearing problems, developmental delay, mental retardation and myopathy. The yeast homolog of GFER, called Erv1p, participates in the disulfide relay system (Twin-cys, Fig. 1), which enables proteins with specific cysteine motifs to be imported into the IMS (Mesecke et al., 2005). The yeast homolog of DPP1 is one of the small Tim proteins (Tim8p), which functions to deliver hydrophobic polytopic membrane proteins for insertion into the IM (Rothbauer et al., 2001). Studies in yeast of mutations of GFER (R194H) (Di Fonzo et al., 2009) and DPP1 (C66W) (Hofmann et al., 2002) that are found in affected individuals provided evidence that defects in mitochondrial protein import were responsible for the disease process. Similarly, individuals with skeletal growth and development disorders were shown to carry a homozygous mutation (N76D) in MAGMAS (mitochondrial-associated granulocyte macrophage colony stimulating factor-signaling gene), a protein belonging to the PAM (presequence translocase-associated motor) component of the TIM23 machinery, which is involved in the delivery of nDNA-encoded proteins into the mitochondrial matrix (Mehawej et al., 2014) (see Fig. 1). A yeast model of this mutation provided a strong indication that the disease was indeed caused by defects in mitochondrial protein import (Mehawej et al., 2014).

#### Diseases caused by defects in metabolite transport

More than ten different MCF (mitochondrial carrier family)-based diseases have been described (Palmieri, 2014), of which several have been molecularly characterized by utilizing yeast. For instance, mitochondrial dysfunction was suspected to underlie high levels in urine of 2-hydroxyglutaric and of Krebs cycle intermediates in individuals displaying agenesis of the corpus callosum (see Box 2) and severe neurodevelopmental problems (Edvardson et al., 2013). Whole-exome sequencing of these individuals revealed two mutational changes (G130D and R282H) in highly conserved positions of the gene encoding the mitochondrial citrate transporter (*SLC25A1*). Subsequent studies revealed that the corresponding mutations in *S. cerevisiae* impaired respiratory growth owing to defects in the transport of citrate (see Box 2) across the IM, leaving little doubt as to their pathogenicity. Similarly, the use of a *S. cerevisiae* model yielded evidence that an A15V mutation in *SLC25A15* found in an individual with hyperornithinemia-hyperammonemia-homocitrullinuria (HHH) syndrome (see Box 2) exerts its deleterious effects by dramatically reducing the transport of ornithine (see Box 2) into mitochondria (Ersoy Tunali et al., 2014). Studies in this yeast have also defined the consequences of several mutations in isoform-1 of the ADP/ATP translocase [adenine nucleotide transporter isoform 1 (ANT1)] that have been associated with various diseases, including autosomal dominant PEO and hypertrophic cardiomyopathy (Kaukonen et al., 2000; Liu and Chen, 2013). Some mutations (e.g. A137D) almost entirely abolish the nucleotide transport activity of the yeast *ANT1* ortholog (*Anc2*), whereas others (A128P, M114P) favor ATP/ATP homo-exchange and thereby compromise oxidative phosphorylation because of a lack of ADP within the organelle. Given the central role of mitochondria in metabolism, yeast are likely to be a powerful tool for continuing to explore the molecular basis of metabolic disorders.

#### Diseases caused by defects in mitochondrial dynamics

With the help of yeast studies, several human diseases have been associated with defects in mitochondrial fusion and fission (Chan, 2012). Approximately 60 mutations in *MNF2*, which encodes a protein involved in OM fusion, have been found in individuals presenting with Charcot-Marie-Tooth disease type 2A (CMT2A), which is characterized by axonal degeneration of peripheral nerves and muscle weakness (Cartoni and Martinou, 2009; Zuchner et al., 2004). Some of these mutations (e.g. I213T) result in fragmented and aggregated mitochondria when introduced in the yeast homologous gene (*FZO1*), whereas others have only negligible effects in yeast, indicating possible mechanistic differences in OM fusion between yeast and humans (Amiott et al., 2009). Defects in IM fusion have been implicated in optic atrophy type 1 (OPA1), a dominantly inherited optic neuropathy that features progressive loss in visual acuity (Votruba et al., 1998). Similar symptoms are found in Leber hereditary optic neuropathy (LHON), which is caused by mutations in mtDNA-encoded CI subunits, suggesting that mitochondrial dysfunction could be involved in OPA1 (Johnston et al., 1979; Kjer et al., 1983). Sequences from the chromosomal region to which OPA1-causing mutations map exhibit homology to a yeast gene that encodes a dynamin-related protein essential for mtDNA maintenance (Msp1p in *Saccharomyces pombe*; Mgm1p in *S. cerevisiae*) (Jones and Fangman, 1992; Pelloquin et al., 1999). This homology helped resolve the structure of the *OPA1* gene and to identify numerous *OPA1* mutations (including frameshift, missense, deletions and insertions) that segregated with the disease, thereby also demonstrating a role for mitochondria in retinal ganglion cell pathophysiology (Alexander et al., 2000). Further studies showed that Mgm1p (OPA1) is a mitochondrial GTPase involved in the fusion of mitochondrial IMs (Ehses et al., 2009; Griparic et al., 2007; Sesaki et al., 2003; Song et al., 2007; Wong et al., 2003). Only one mutation affecting mitochondrial fission has been associated with human disease thus far: encephalopathy with optic atrophy caused by the A395D variant of the dynamin-like DRP1 protein (Waterham et al., 2007). Its yeast homolog (Dnm1p) localizes and oligomerizes at restricted sites on the surface of mitochondria, suggesting a dynamin-like contractile mechanism for mitochondrial fission (Fukushima et al., 2001; Ingerman et al., 2005; Mears et al., 2011). Modeling this pathogenic mutation in yeast prevented the oligomerization of Dnm1p (DRP1) owing to its decreased hydrolysis of GTP, suggesting this as a potential mechanism of disease.

#### Diseases caused by defects in mitochondrial protein quality control

Hereditary spastic paraplegia (HSP) constitutes a genetically and clinically heterogeneous group of neurodegenerative disorders characterized mainly by progressive lower-limb weakness, spasticity and decreased vibratory sense (Harding, 1983). In 1998, an autosomal recessive form of HSP was associated with mutations in a gene (*SPG7*) encoding a protein (paraplegin) with strong similarities to the two homologous subunits of the yeast m-AAA protease [called Afg3 (Yta10) and Rca1 (Yta12)] (Casari et al., 1998). This protease controls the formation of the respiratory chain complexes (Arlt et al., 1998). Like its cognate yeast proteins, paraplegin was shown to localize to mitochondria, and loss-of-function mutations led to ragged-red fibers, a hallmark of mitochondrial disorders, and to OXPHOS defects (Casari et al., 1998). A homology search yielded two paraplegin-related genes, *AFG3L2* and *YME1L1*, presumed to be the human orthologs of the yeast genes encoding Afg3p and the protein that constitutes the i-AAA protease (Yme1p), respectively (Banfi et al., 1999; Coppola et al., 2000). Paraplegin co-assembles with AFG3L2, and this interaction is required for the proper expression of CI (Atorino et al., 2003). Mutations in *AFG3L2* cause autosomal dominant spinocerebellar ataxia type 28 (SCA28), a neurological disorder caused by Purkinje-cell degeneration (Di Bella et al., 2010). The human SPG7-AFG3L2 complex rescued yeast strains lacking Rca1p and Afg3p, demonstrating functional conservation and providing a simple assay to evaluate the functional consequences of *SPG7* and *AFG3L2* mutations found in affected individuals (Bonn et al., 2011; Di Bella et al., 2010). Many of these mutations were unable to restore respiratory competence in m-AAA-deficient yeast strains, providing additional evidence for their deleterious nature in humans (Di Bella et al., 2010).

#### Diseases caused by defects in cardiolipin synthesis and remodeling

Cardiolipin (CL) is a mitochondrial-specific lipid that mostly localizes to the IM, where it is synthesized (Schlame and Haldar, 1993; Schlame et al., 2000). Studies in yeast have defined how CL is synthesized and remodeled to maintain a homogenous and highly unsaturated acyl-chain composition, and how mitochondria are influenced by defects in these processes (Claypool, 2009; Joshi et al., 2009; Mileykovskaya and Dowhan, 2009). Yeast strains that fail to synthesize CL respire poorly and can neither organize the mitochondrial energy-transducing enzymes into supercomplexes (i.e. the respirasome) nor promote their association with the ADP/ATP translocase (ANT), indicating that CL is required for the formation and/or stability of these multi-complex assemblies. As a consequence, the mitochondrial membrane potential (ΔΨ) is decreased, which negatively affects the import of numerous proteins into the matrix and IM (Joshi et al., 2009). Moreover, CL interacts with components involved in IM fusion (Mgm1p) and mitochondrial fission (Dnm1p), and the loss of these interactions possibly contributes to the abnormal mitochondrial morphologies observed in yeast strains lacking CL (Ban et al., 2010; DeVay et al., 2009; Montessuit et al., 2010).

Given the importance of CL for mitochondrial structure and function, it is not surprising that defects in the synthesis and remodeling of this lipid are associated with various disorders (Chicco and Sparagna, 2007). One such disease, Barth syndrome (BTHS), is caused by mutations in the human gene *TAZ*, which encodes tafazzin, an acyl transferase involved in the remodeling of CL (Barth et al., 1983; Schlame and Ren, 2006). BTHS is an X-linked disease exhibiting cardiac and skeletal myopathies, delayed growth until puberty, and increased susceptibility to bacterial infections due to cyclic neutropenia (see Box 2). Individuals with BTHS are characterized by pleiotropic respiratory defects (Barth et al., 1996), possibly because of impaired respirasome stability (McKenzie et al., 2006), have low levels of CL, and accumulate monolysocardiolipin (MLCL), an intermediate in the CL remodeling pathway that lacks an acyl chain, in various tissues and cells (Schlame et al., 2003; Valianpour et al., 2005). Yeast strains lacking Taz1p (*taz1Δ*), the homolog of tafazzin, also accumulate MLCL with a concurrent decrease in CL (Claypool et al., 2006, 2011; Gu et al., 2004; Testet et al., 2005; Vaz et al., 2003). These strains display a slow growth phenotype on respiratory substrates at 37°C and decreased respirasome stability (Brandner et al., 2005; Gu et al., 2004; Vaz et al., 2003), providing a simple assay to test the functional consequences of mutations found in individuals with BTHS. Most of these mutations were found to impair CL expression in yeast owing to mislocalization in the matrix or rapid degradation of the mutated protein (by the i-AAA protease), confirming their deleterious nature (Claypool et al., 2006, 2011). Yeast Taz1p assembles into distinct high-molecular-weight complexes containing various subunits of ATP synthase and CIII, ANT, and as-yet-unidentified binding partners (Claypool et al., 2008). Future studies should help to reveal how Taz1p (tafazzin) influences mitochondrial functions in normal and pathological conditions.

## Molecular insights from yeast models with translational potential

As discussed below, several approaches in yeast have been used to unravel potential strategies for treating mitochondrial disorders. This model organism offers simple readouts, such as the common respiratory growth defect observed in yeast models of mitochondrial disease, to enable large-scale screens for genetic suppressors (Box 4) and chemicals able to rescue mitochondrial dysfunction. Even when mitochondrial dysfunction is severe enough to abolish respiratory growth, yeast offers the unique advantage that such mutants can be kept alive and propagated on fermentable substrates for their use in suppressor screens. Forward chemical genetic approaches can also be performed in yeast to uncover potential chemical targets (St Onge et al., 2012).

Box 4. **Genetic suppression**

In genetic suppression, a mutant phenotype is reversed by the effects of a mutation at a locus distinct to that of the original mutation. The suppressor mutation can be located: (1) within the same gene as the primary (target) mutation, at the same or at a different nucleotide position (intragenic suppression); (2) in a different gene of the same genome (intergenic suppression); or (3) in the case of a mitochondrial phenotype, within another genome (intergenomic suppression), since two different genomes control mitochondrial function.

### New insights from metabolic suppression studies

A popular suppressor genetics method in yeast aims to identify genes that, when overexpressed, rescue a mutation in another gene. This can be done using libraries of yeast genes cloned into multicopy plasmids. Unexpectedly, overexpressing the gene encoding Odc1p, a mitochondrial carrier that transports Krebs cycle intermediates, compensates for the lack of a protein (Fmc1p) involved in the assembly of CV (Schwimmer et al., 2005). Although the CV assembly remained defective, artificially increasing the levels of Odc1p (by tenfold) in *fmc1Δ* yeast substantially stimulated respiration and ATP production through substrate-level phosphorylation in mitochondria. The overexpression of Odc1p also rescued mutant strains lacking the yeast homolog of the human *MPV17* gene (*SYM1*) implicated in diseases characterized by mtDNA loss in the liver (Dallabona et al., 2010). Taken together, these studies signify that metabolic suppression is a promising approach for generating therapeutic leads for mitochondrial diseases.

### Suppressors of disease-causing mt-tRNA mutations

Given the sequence and structural similarities between some human and yeast mt-tRNAs, yeast has been used to model pathogenic base substitutions in these molecules, notably in tRNA^Leu(UUR)^, which attaches the amino acid leucine (Leu) (Montanari et al., 2008). Some of these mutations severely affect yeast respiratory growth, providing a phenotype to use in multicopy suppressor gene screens. Several factors involved in mitochondrial protein synthesis have been identified using this strategy, including the translation factor EF-Tu (TUFM in humans) and various (cognate and non-cognate) aminoacyl tRNA synthetases (aa-RSs; see Box 2) (Feuermann et al., 2003; Montanari et al., 2010). The suppressor activity of these factors was also observed in human cells carrying similar mutations (Park et al., 2008; Sasarman et al., 2008; Rorbach et al., 2008; Li and Guan, 2010). Interestingly, after the introduction of point mutations that inactivate their tRNA charging function, aa-RSs maintained their suppressor activity, which indicates that the mutated mt-tRNAs recover their functionality likely owing to chaperone-like RNA-protein interactions (Francisci et al., 2011). Short regions of less than 70 amino acids near the C-terminus of aaRSs were sufficient to improve mitochondrial translation, both in yeast and human cells with defective mt-tRNAs. These findings hold promise for the development of peptide-based therapies against diseases induced by mutations in mt-tRNAs.

### Genetic suppressors of BCS1-based disorders

Yeast has also been used to explore potential mechanistic strategies to rescue Björnstad and GRACILE (BCS1-based) syndromes (Ostojic et al., 2013). The former is a relatively mild disease characterized by twisted hairs (pili torti) and hearing problems (Hinson et al., 2007), whereas the latter is a much more severe disorder characterized by growth retardation, aminoaciduria, cholestasis (see Box 2), iron overload, lactic acidosis and early death, sometimes before birth (Visapaa et al., 2002). The BCS1 protein belongs to the large and evolutionarily conserved AAA protein family, characterized by the presence of a typical AAA region involved in ATP binding and hydrolysis. It is required to incorporate Rieske iron sulfur protein (Rip1) into CIII (Nobrega et al., 1992; Wagener et al., 2011). When modeled in yeast Bcs1p, several pathogenic mutations in the AAA region of human BCS1 were shown to prevent respiration in yeast owing to a blockade in the assembly of CIII (Ostojic et al., 2013). Unexpectedly, Rip1 assembly was restored in these *bcs1* yeast mutants owing to secondary mutations that reduce the ATP hydrolytic activity of CV while maintaining a sufficient level of ATP synthesis to sustain respiratory growth. It was reasoned that by reducing the ATP hydrolysis of CV, the suppressors increase the organellar concentration of ATP and thereby enable the mutated BCS1 protein to reach sufficient ATP hydrolytic activity (Ostojic et al., 2013). This hypothesis was supported by *in vitro* assays showing that BCS1 hydrolytic activity returned to normal levels by increasing the concentration of ATP (Ostojic et al., 2013). This genetic interaction between BCS1 and ATP synthase suggests that the AAA region serves not only to provide the BCS1 protein with the energy required to accomplish its chaperone function, but also as a sensor of the ATP:ADP ratio in mitochondria to adjust the production of CIII according to the cell's metabolic state. This study elegantly identified the intra-mitochondrial pool of adenine nucleotides as a potential target for improving the condition of patients suffering from defects in BCS1, and possibly in other AAA proteins involved in mitochondrial biogenesis (such as m-AAA).

### Genetic suppressors of ANT-based disorders

Given the importance of ANT (adenine nucleotide transporter) for mitochondrial physiology, not surprisingly, mutations or altered expression of this protein result in various human diseases, such as adPEO (autosomal dominant progressive external ophthalmoplegia), cancer, FSHD (facioscapulohumeral muscular dystrophy) and Senger's syndrome, which is characterized by cardiac hypertrophy, mitochondrial myopathy, cataracts and lactic acidosis (Liu and Chen, 2013). Studies in yeast have suggested that human pathogenic mutations in ANT1 (isoform 1 of ANT) might not only cause the defective exchange of adenine nucleotides across the mitochondrial IM but also induce mitochondrial biogenesis defects, thereby severely compromising yeast cell viability (even in fermentable media) owing to the partial uncoupling of the mitochondrial IM (Liu and Chen, 2013; Wang et al., 2008). Interestingly, mutations and chemicals that reduce cytosolic protein synthesis substantially improve the viability of yeast models of ANT1-based diseases and suppress some of their associated mitochondrial phenotypes, such as the loss of mtDNA integrity. These findings indicate that mutations in ANT1 can lead to general cellular protein stress due to a reduced capacity of the mitochondria to import nDNA-encoded proteins. They highlight cytosolic protein synthesis as a potential therapeutic pathway for ANT1-based diseases and possibly for other disorders that affect the delivery of proteins into the organelle either directly or indirectly by altering the proton pumping activity or the coupling efficiency of the OXPHOS system.

### Genetic suppressors of mtDNA maintenance defects

Genetic suppressors have uncovered potential intervention points for diseases caused by decreased mtDNA content. One approach used mutations in various cellular systems (e.g. ANT, F_1_-ATPase) that convert *S. cerevisiae* into a ‘petite-negative’ yeast unable to survive without mtDNA (Chen and Clark-Walker, 2000). Interestingly, genetic perturbations in nutrient-responsive signaling pathways that restored petite-positivity proved to increase the health of yeast cells lacking mtDNA (Garipler and Dunn, 2013; Garipler et al., 2014). Other studies have shown that increasing the availability of mitochondrial dNTP [a well-known limiting factor in mtDNA replication (Lebedeva and Shadel, 2007)], either by overexpressing the large subunit of ribonucleotide reductase (Rnr1p) or by deleting a gene encoding a protein inhibitor of Rnr1p [Sml1p (suppressor of Mec1 lethality)], significantly suppressed the instability of the mitochondrial genome in yeast strains bearing mutations in the mitochondrial DNA polymerase gene *mip1* (Baruffini et al., 2006; Zhao et al., 1998).

## Pharmacological suppressors

Yeast has been proposed as a pharmacological model to identify drugs that are active against mitochondrial diseases (di Rago et al., 2007; Schwimmer et al., 2006). Although this approach is fairly recent, a number of fruitful studies have been described, which we briefly review here.

**Fig. 4.**
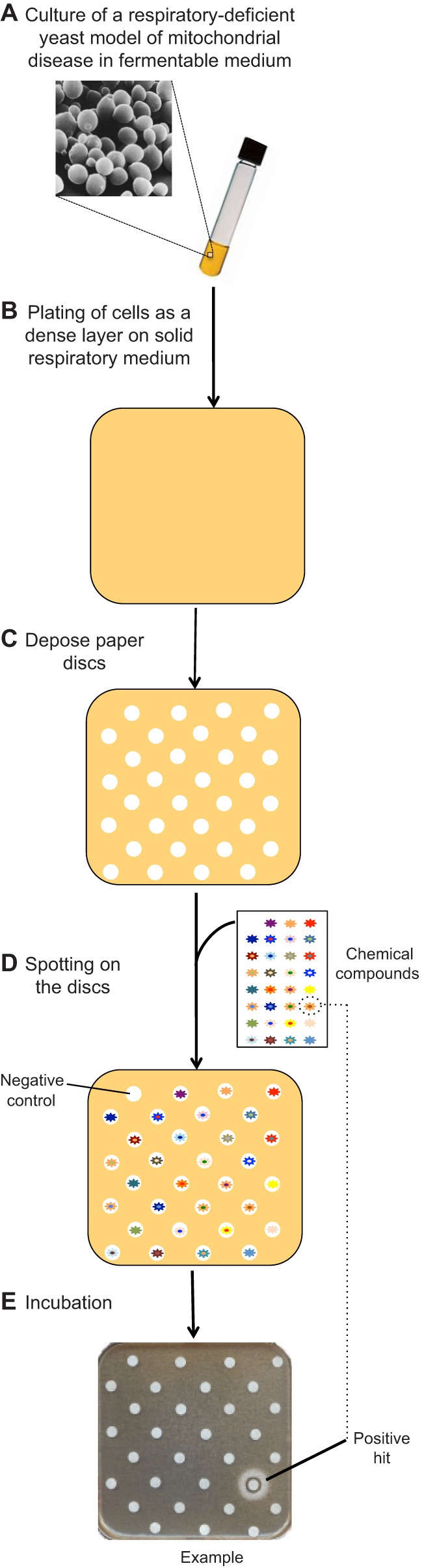
**A yeast-based assay to identify drugs that are active against mitochondrial disorders.** (A) A respiratory-deficient yeast model of a mitochondrial disease is grown in glucose. (B) Subsequently, yeast cells are spread onto a solid medium containing a non-fermentable substrate (glycerol), on which they grow very poorly. (C) Small sterile filters are placed on the agar surface and (D) spotted with compounds from a chemical library; the plate is then incubated for several days. (E) After incubation, active drugs that improve mitochondrial function in the yeast disease model result in the appearance of a halo of enhanced growth around the corresponding filters.

The first such study used a yeast model (*fmc1Δ*, described above) that phenotypically resembles diseases caused by deficiency in fully assembled ATP synthase (Couplan et al., 2011). The *fmc1Δ* cells were spread on solid respiratory medium, on which they grow very slowly, and were then exposed to filters spotted with individual drug compounds. After a few days of incubation, active compounds were identified by the appearance of a halo of enhanced growth around the corresponding filters (see Fig. 4 for an example). This method allows, in one simple experiment, the testing of numerous compounds across a large range of concentrations, owing to their diffusion in the growth medium – a powerful design for assessing when a drug is active at low concentrations while toxic at higher ones. This screen used, among others, the Prestwick Chemical Library, a collection of drugs with high bioavailability and for which toxicity studies have already been carried out in humans; therefore, active compounds from this library can directly enter drug optimization programs.

Among positive hits that improved respiratory growth of *fmc1Δ* yeast was chlorhexidine (CH), a well-known antiseptic (Couplan et al., 2011). This drug had a remarkable suppressor activity in *fmc1Δ* yeast, with a substantial (more than twofold) increase in mitochondrial respiration and ATP synthesis due to a better expression of OXPHOS enzymes, which was not observed in wild-type yeast treated with CH. Additionally, upon treatment with CH, the *fmc1Δ* cells recovered the ability to form mitochondrial cristae and no longer displayed inclusion bodies formed by the aggregation in the mitochondrial matrix of unassembled subunits of CV (Couplan et al., 2011). In a secondary screen, CH was also shown to rescue yeast models of diseases [NARP (neuropathy ataxia retinitis pigmentosa), MILS (maternally inherited Leigh syndrome)] caused by mutations in the mitochondrial *ATP6* gene. This drug was next tested in human cybrid (cytoplasmic hybrid; Box 2) cell lines that were nearly homoplasmic (Box 2) for one of these mutations (T8993G), using a glucose-deprived medium to force the cells to rely on OXPHOS rather than glycolysis (Box 2). A clear dose-dependent improvement of NARP cybrid survival was observed, whereas the growth of wild-type cybrids remained unchanged in the presence of CH.

Another drug that markedly improved the respiratory growth of *fmc1Δ* yeast, and which also proved therapeutic in patient-derived T8993G cybrids, was sodium pyrithione (NaPT) (Aiyar et al., 2014). The pathway(s) through which NaPT rescues ATP synthase deficiencies were investigated by systematic chemical-genomic profiling using the yeast genome-wide deletion collection. In this approach, the pronounced sensitivity of haploinsufficient, heterozygous deletion mutants to a chemical can indicate cellular functions and proteins involved in the chemical's mechanism of action. The most sensitive mutants included *tim17Δ*/*TIM17* and *tim23Δ*/*TIM23*, which involve essential components of the highly conserved TIM23 translocase complex of the mitochondrial IM (Dolezal et al., 2006; Hoogenraad et al., 2002) (Fig. 1). The sensitivity of these mutants to NaPT was far greater than observed for many previously profiled compounds, indicating that the chemical-genetic interaction between NaPT and TIM23 is highly specific. *In vitro* assays revealed that NaPT partially compromised the import of proteins by TIM23 machinery into the matrix, whereas its lateral sorting activity to the IM was enhanced. The therapeutic effects of modulating TIM23-mediated import in this precise manner were confirmed by overexpressing the regulatory subunit Tim21p, which affects import in a similar way to NaPT (Chacinska et al., 2009; Popov-Celeketic et al., 2008). Tim21p overexpression substantially restored the respiratory capacity of *fmc1Δ* yeast through improved activity and expression of electron transport chain (ETC; Box 2) complexes and ATP synthase, and overexpression of its human homolog TIM21 also rescued human T8993G cybrids. Although a general inhibition of TIM23 would be detrimental, these findings suggest that a slight modulation of its activity could be beneficial in the context of mitochondrial dysfunction. This study also fits with recent reports indicating that downregulating TIM23-mediated protein import can be used as a stress response to maintain protein homeostasis in mitochondria (Nargund et al., 2012; Rainbolt et al., 2013). Because of the central role of the TIM23 pathway in mitochondrial function and biogenesis, its therapeutic potential could possibly extend to other types of mitochondrial dysfunction.

A similar screening assay was developed for Friedreich’s ataxia (FRDA), which is a common autosomal recessive degenerative disease resulting from a GGA trinucleotide expansion within an intron (Box 2) of a nuclear gene encoding a protein (frataxin) that controls mitochondrial iron homeostasis (Rotig et al., 1997). Using a yeast strain lacking the orthologous gene, *YFH1* (yeast frataxin homolog) (Foury and Cazzalini, 1997), a number of potential compounds for the treatment of FRDA that function via an as-yet-unknown mechanism were identified (Cotticelli et al., 2012). Finally, a recently published yeast-based assay was employed to screen for small molecules that increase the mitochondrial membrane potential and cellular ATP levels (Montague et al., 2014). Fourteen positive hits were isolated from a collection of 13,680 compounds, of which several were able to increase ATP levels in hepatocytes and fibroblasts. Genomic and mitochondrial proteomic analyses indicate that the drug response in the human cells involves key factors controlling metabolic functions such as PGC-1α (peroxisome proliferator-activated receptor gamma coactivator 1-alpha), which is an animal transcriptional coactivator that regulates genes involved in energy metabolism. Taken together, these studies validate the use of yeast-based models for effective high-throughput screening approaches aimed at identifying drugs with the potential to restore mitochondrial function and to treat mitochondrial disorders.

## Conclusions

Although considerable progress in understanding mitochondrial function has been made during the last decades, much remains to be learned about mitochondrial processes and components, their regulation and their interplay with the rest of the cell. Owing to the exceptionally thorough annotation of its genome, its tractability for manipulating mitochondrial and nuclear genes, and the high conservation of mitochondrial function, *S. cerevisiae* will continue to be an essential model system for this major research challenge.

Expanding our comprehension of mitochondrial biology further will be instrumental to better define human diseases caused by mitochondrial dysfunction. Considering the huge complexity of human mitochondria, with approximately 1500 proteins, it is likely that the list of mitochondrial diseases (<200 have been described so far) will rapidly increase. Although some functions fulfilled by human mitochondria do not exist in yeast (for example, calcium storage and apoptosis), human and yeast mitochondrial proteomes are so similar that yeast is very well-suited to determine the primary effects on mitochondrial energy transduction and physiology of disease-linked mutations (Schwimmer et al., 2006).

Those diseases associated with mtDNA variants, the list of which is rapidly expanding, are particularly challenging to study owing to factors like heteroplasmy (Box 2), complex inheritance, variable penetrance and interactions with (e.g. nuclear) modifier genes, which makes it difficult to verify their pathogenicity, let alone understand how they lead to disease. The ability to introduce mtDNA mutations in a defined nuclear genetic background and the absence of stable heteroplasmy in *S. cerevisiae* allows human mtDNA variants to be studied in isolation, which has often proven useful in elucidating the mechanistic basis of their pathogenicity. Developing mtDNA genetic tools in the yeast *Y. lipolytica* would be of great interest to model pathogenic mutations of mtDNA that affect the CI, an essential energy-transducing system not present in *S. cerevisiae* that is often implicated in human disorders (see Fig. 2).

One of the most devastating aspects of mitochondrial diseases is the dearth of effective therapeutic strategies. Here also, yeast could provide a tremendous help by enabling straightforward and fairly easy selection of correcting mechanisms, by way of genetic suppressors and chemical screening. Although fairly recent, this approach has already pointed to the therapeutic potential of peptide-based therapies, metabolic suppression or bypasses, and targeting the regulation of pathways not necessarily implicated in the disease. Such revelations underscore the power of yeast models and system approaches for unearthing novel and otherwise unpredictable candidate intervention points for the treatment of mitochondrial disorders. As a unicellular organism, yeast cannot be used to model a disease at the scale of an organ or an intact complex multicellular organism. However, modeling mitochondrial dysfunction at the multi-organ level is now possible in invertebrate (e.g. *Caenorhabditis elegans*) as well as vertebrate (e.g. *Danio rerio*) animals, in which, like in yeast, most biological processes involved in mitochondrial function are conserved. These systems offer a way to test whether molecular findings can be moved from yeast into translational research.

## Supplementary Material

Supplementary Material
