# Supplementary material for: Yeast as a system for modeling mitochondrial disease mechanisms and discovering therapies
Source: Dis Model Mech. 2015 Jun 1;8(6):509–26. doi: 10.1242/dmm.020438 (PMC4457039; doi:10.1242/dmm.020438)
Supplement: Supplementary Material [file supp_8_6_509__index.html]

Yeast as a system for modeling mitochondrial disease mechanisms and discovering therapies — Supplementary Material 

# Yeast as a system for modeling mitochondrial disease mechanisms and discovering therapies

## DMM020438 Supplementary Material

**Files in this Data Supplement:**

- **Supplementary Material**
