# Supplementary material for: Yeast as a system for modeling mitochondrial disease mechanisms and discovering therapies
Source: Dis Model Mech. 2015 Jun 1;8(6):509–26. doi: 10.1242/dmm.020438 (PMC4457039; doi:10.1242/dmm.020438)
Supplement: Supplementary Material [file supp_8.6.509_DMM020438.pdf]

H.s. genes	*S.c.* genes	Molecular function within mitochondria	Mitochondrial Diseases	% id	*H.s.* /*S.c.* protein length
Nuclear genes					
**Respiratory Complex II & TCA cycle**					
SDHA	*SDH1*	*flavoprotein subunit of succinate dehydrogenase*	Leigh syndrome	63	664/660
SDHB	*SDH2*	*Fe/S subunit of succinate dehydrogenase*	paraganglioma	63	280/266
SDHC	*SDH3*	*membrane anchor subunit of succinate dehydrogenase*	paraganglioma	23	169/198
SDHD	*SDH4*	*membrane anchor subunit of succinate dehydrogenase*	paraganglioma	16	159/181
SDHAF1	*YDR379C-A (SDH6)*	*involved in SDH assembly*	leukoencephalopathy	20	115/79
SDHAF2	*EMI5 (SDH5)*	*SDHA flavination*	paraganglioma	35	166/162
DLAT	*LAT1*	*Dihydrolipoamide acetyltransferase component (E2) of the PDC*	hypotonia, neurological syndrome	30	647/482
DLD	*DLD1*	*D-lactate dehydrogenase*	encephalopathy	13	509/587
SUCLA2	*LSC2*	*Beta subunit of succinyl-CoA ligase*	encephalomyopathy, mtDNA depletion syndrome	42	405/427
SUCLG1	*LSC1*	*Alpha subunit of succinyl-CoA ligase*	encephalomyopathy, mtDNA depletion syndrome	53	346/329
PDHB	*PDB1*	*E1 beta subunit of the pyruvate dehydrogenase (PDH) complex*	neurological syndrome	56	359/366
PDHX	*PDX1*	*E3-binding protein of the mitochondrial pyruvate dehydrogenase complex*	neurological syndrome	18	501/410
ACO2	*ACO1*	*aconitase*	cerebellar degeneration	66	778/780
IDH2	*IDH2*	*subunit of mitochondrial NAD(+)-dependent isocitrate dehydrogenase*	Maffucci syndrome, encephalopathy	17	452/362
IDH3B	*IDH1*	*subunit of mitochondrial NAD(+)-dependent isocitrate dehydrogenase*	retinitis pigmentosa	43	385/360
OGDH	*KGD1*	*subunit of the mitochondrial alpha-ketoglutarate dehydrogenase complex*	metabolic acidosis, hypoglycemia	44	1023/1014
FH	*FUM1*	*fumarase; converts fumaric acid to L-malic acid in the TCA cycle*	encephalopathy	62	510/488
PDHA1	*PDA1*	*Link between TCA and OXPHOS, catalyzing conversion of pyruvate in acetyl-CoA*	Leigh syndrome, X-linked; Pyruvate dehydrogenase E1-alpha deficiency	44	390/420
**Respiratory Complex III**					
UQRC2	*COR2*	*subunit of ubiquinol cytochrome-c reductase (complex III)*	metabolic acidosis	20	453/368
UQCRB	*QCR7*	*subunit of ubiquinol cytochrome-c reductase (complex III)*	metabolic acidosis	31	111/127
UQCRQ	*QCR8*	*subunit of ubiquinol cytochrome-c reductase (complex III)*	neurological defect	18	82/94
CYC1	*CYT1*	*cytochrome c1, catalytic subunit of complex III*	metabolic acidosis	45	325/309
BCS1	*BCS1*	*protein required for the assembly of the FeS subunit into complex III*	encephalopathy with hepatic failure, Gracile syndrome	45	419/456
HCCS	*CYC3*	*cytochrome c heme lyase, attaches heme to apo-cytochrome c*	microphtalmia	31	268/269
CYCS	*CYC1, CYC7*	*cytochrome c*	hyperglycemia, thrombocytopenia	59	105/109
UQCC2	*CBP6*	*protein required for translation of the COB mRNA*	mental retardation	13	126/162
LYRM7 (MZM1L)	*MZM1*	*protein required for the assembly of the FeS subunit into complex III*	encephalopathy	21	104/123
**Respiratory Complex IV**					
COX4I2	*COX5*	*subunit of cytochrome c oxidase (complex IV)*	exocrine pancreatic deficiency	15	171/153
COX6B1	*COX12*	*subunit of cytochrome oxidase (complex IV)*	cardioencephalomyopathy	40	86/83
COX10	*COX10*	*heme A:farnesyltransferase*	Leigh syndrome, cardiomyopathy	28	443/462
COX15	*COX15*	*protein required for the hydroxylation of heme O to form heme A*	Leigh syndrome, cardiomyopathy	33	410/486
COX20	*COX20*	*required for proteolytic processing of Cox2p and its assembly into cytochrome c oxidase*	hypotonia, cerebellar ataxia	12	118/205
SCO1	*SCO1*	*deliver copper to complex IV*	Leigh syndrome, cardiomyopathy	30	301/295
SCO2	*SCO2*	*transfer of Cu or cysteine reduction in Cox2p*	Leigh syndrome, cardiomyopathy	27	266/301
SURF1	*SHY1*	*involved in complex IV assembly*	Leigh syndrome	22	300/389
COX14-(C12ORF62)	*COX14*	*involved in translational regulation of Cox1p and assembly of complex IV*	metabolic acidosis	13	57/70
PET100	*PET100*	*protein required for the assembly of complex IV*	Leigh syndrome	17	73/111
COA5-(C2ORF64)	*PET191*	*protein required for assembly of cytochrome c oxidase*	cardiomyopathy	18	74/108
LRPPRC	*PET309*	*specific translational activator for the COX1 mRNA*	Leigh syndrome	11	1394/965

H.s. genes	*S.c.* genes	Molecular function within mitochondria	Mitochondrial Diseases	% id	*H.s.* /*S.c.* protein length
TACO1	*YGR021w*	*unknown*	Leigh syndrome, cardiomyopathy	23	297/290
ATP5A1	*ATP1*	*alpha subunit of the F1 sector of mitochondrial F1F0 ATP synthase*	encephalopathy	69	553/545
ATP5E	*ATP15*	*epsilon subunit of the F1 sector of mitochondrial F1F0 ATP synthase*	cardiomyopathy, neuropathy	24	51/62
ATPAF2	*ATP12*	*assembly of F1 portion of ATP synthase*	cardiomyopathy, metabolic acidosis	18	328/289
**Carriers**					
ANT1, SLC25A4	*AAC3, AAC2, AAC1*	*ATP/ADP carrier*	ophthalmoplegia, cardiomyopathy	49	298/307
SLC25A3	*PIC2*	*inorganic phosphate carrier*	sideroblastic anemia, cardiomyopathy	38	362/300
SLC25A1	*CTP1*	*citrate carrier*	encephalopathy	34	311/299
SLC25A12, A23, A22	*AGC1*	*acts both as a glutamate uniporter and as an aspartate-glutamate exchanger*	epileptic encephalopathy	25	678/902
SLC25A15	*ORT1*	*exports ornithine from mitochondria as part of arginine biosynthesis*	HHH syndrome	38	301/292
SLC25A19	*TPC1*	*mediates uptake of the essential cofactor thiamine pyrophosphate (ThPP) into*	microcephaly	25	320/314
SLC25A20	*CRC1*	*carnitine transporter*	neuropathy, cardiopathy, liver dysfunction	29	301/327
SLC25A38	*HEM25*	*Iron transporter, mediates Fe2+ transport across inner mito membrane*	sideroblastic anemia	27	304/314
**CoQ biosynthesis**					
ADCK3 (CABC1)/ADCK	*COQ8 (ABC1)*	*protein required for ubiquinone biosynthesis*	cerebellar ataxia and seizures	30	647/501
COQ2	*COQ2*	*para hydroxybenzoate polyprenyl transferase*	Leigh syndrome, nephropathy, Nephrotic syndrome through CoQ10 biosynthesis disruption	35	371/372
COQ6	*COQ6*	*putative flavin-dependent monooxygenase*	nephropathy	29	468/479
COQ9	*COQ9*	*protein required for ubiquinone biosynthesis*	nephropathy, cardiomyopathy, encephalopathy	19	318/260
PDSS1, PDSS2	*COQ1*	*Hexaprenyl pyrophosphate synthetase*	encephaloneuropathy, Leigh syndrome, nephropathy	25	415/473
**Phospholipids**					
TAZ	*TAZ1*	*lyso-phosphatidylcholine acyltransferase*	Barth syndrome (cardiomyopathy and cyclic neutropenia)	19	292/381
**FeS biogenesis - ROS response**					
ABCB7	*ATM1*	*ATP-binding cassette (ABC) transporter: exports iron-sulfur (Fe/S) clusters to the cytosol*	anemia, ataxia	41	752/690
ALAS2	*HEM1*	*5-aminolevulinate synthase; catalyzes the first step in the heme biosynthetic pathway*	anemia, protoporphyria	32	587/548
FXN	*YFH1*	*iron chaperone, formation of Fe-S clusters*	Friedreich Ataxia	25	210/174
ISCU	*ISU1*	*scaffolding function during assembly of iron-sulfur clusters,*	myopathy	55	167/165
NFU1	*NFU1*	*protein involved in iron metabolism in mitochondria*	encephalopathy	29	254/256
GLRX5	*GRX5*	*glutathione-dependent oxidoreductase*	sideroblastic anemia	34	157/150
SFXN4	*FSF1*	*predicted to be an alpha-isopropylmalate carrier*	growth retardation, hypotonia	22	337/327
BOLA3	*AIM1*	*unknown*	encephalopathy, cardiomyopathy	24	107/118
IBA57 (c1orf69)	*IBA57*	*involved in the incorporation of iron-sulfur clusters into mitochondrial aconitase-type proteins*	encephalomyopathy	18	356/497
LYRM4	*ISD11*	*iron-sulfur cluster biogenesis factor*	neonatal lactic acidosis	37	91/94
NFS1	*NFS1*	*iron-sulfur cluster assembly*	lactic acidemia and hypotonia	56	457/497
FDX1L	*YAH1*	*iron-sulfur cluster biogenesis*	myopathy	33	183/172
**DNA, dNTP synthesis**					
POLG	*MIP1*	*mtDNA polymerase*	ataxia, ophthalmoplegia, encephalopathy, Alpers syndrome	26	1239/1254
RRM2B §	*TYMP*	*ribonucleotide-diphosphate reductase (RNR), small subunit*	ophthalmoplegia, encephalomyopathy, Kearns-Sayre syndrome	55	351/399
MPV17	*SYM1*	*unknown*	neurohepatopathy	26	176/197
TYMP §	*ADO1*	*cytosolic thymidine phosphorylase*	mitochondrial neurogastrointestinal encephalomyopathy (MNGIE)	35	341/482

H.s. genes	*S.c.* genes	Molecular function within mitochondria	Mitochondrial Diseases	% id	*H.s.* /*S.c.* protein length
**Translation**					
MRPL12	*MNP1*	*ribosomal protein of the large subunit of the mitochondrial ribosome*	Growth retardation, neurological distress	32	198/194
MRPS16	*MRPS16*	*ribosomal protein of the small subunit*	Metabolic acidosis	27	137/121
MRPL3	*MRPL9*	*ribosomal protein of the large subunit*	cardiomyopathy	26	348/269
MRPL44	*MRPL3*	*ribosomal protein of the large subunit*	cardiomyopathy	13	332/390
AARS2	*ALA1*	*mitochondrial alanyl-tRNA synthetase*	cardiomyopathy	37	985/983
DARS2	*MSD1*	*mitochondrial aspartyl-tRNA synthetase*	leukoencephalopathy	30	645/658
EARS2	*MSE1*	*mitochondrial glutamyl-tRNA synthetase*	leucoencephalopathy	33	523/536
FARS2	*MSF1*	*mitochondrial phenylalanyl-tRNA synthetase*	Alpers syndrome	33	451/469
GARS	*GRS1, GRS2*	*cytoplasmic and mitochondrial glycyl-tRNA synthase*	neuropathy, Charcot-Marie-Tooth disease	39	739/690
HARS2	*HTS1*	*mitochondrial histidine-tRNA synthetase*	Perrault syndrome	40	506/546
KARS	*MSK1*	*cytoplasmic and mitochondrial lysine-tRNA synthetase*	neuropathy, Charcot-Marie-Tooth disease, deafness	28	597/576
LARS2	*NAM2*	*mitochondrial leucyl-tRNA synthetase*	Perrault syndrome 4	33	903/894
MARS2	*MSM1*	*mitochondrial methionyl-tRNA synthetase*	Ataxia, ARSAL syndrome	32	593/575
RARS2	*MSR1*	*mitochondrial arginyl-tRNA synthetase*	encephalopathy, pontocerebellar hypoplasia	35	578/643
SARS2	*DIA4*	*mitochondrial seryl-tRNA synthetase*	HUPRA syndrome	29	518/446
TARS2	*MST1*	*mitochondrial threonyl-tRNA synthetase*	idiopatic inflammatory disease	25	718/462
VARS2	*VAS1*	*mitochondrial and cytoplasmic valyl-tRNA synthetase*	hypotonia	38	1063/1104
YARS2	*MSY1*	*mitochondrial tyrosyl-tRNA synthetase*	MLASA syndrome	30	477/492
MTFMT	*FMT1*	*methionyl-tRNA formyltransferase*	developmental delay, optic atrophy	21	389/401
GFM1	*MEF1*	*mitochondrial elongation factor involved in translational elongation*	hepatoencephalopathy	51	751/761
TUFM	*TUF1*	*mitochondrial translation elongation factor Tu*	hepatoencephalopathy, cardiomyopathy	54	452/437
TSFM	*TSF1**	*mitochondrial translation elongation factor Ts*	encephalomyopathy, cardiomyopathy, liver failure	21	325/299
C12orf65	*YLR281C*	*unknown*	spastic paraplegia, Leigh syndrome	23	155/166
PUS1	*PUS1, PUS2*	*tRNA:pseudouridine synthase*	myopathy, MLASA syndrome	25	427/544
MTO1	*MTO1*	*perform the 5-carboxymethylaminomethyl modification of the wobble base in mitochondrial tRNAs*	cardiomyopathy	42	717/669
TRMU	*SLM3 (MTO2)*	*tRNA-specific 2-thiouridylase*	myopathy, deafness	25	421/417
RMND1	*YDR282C*	*unknown*	encephalopathy	17	449/414
CPS1	*URA2*	*bifunctional carbamoylphosphate synthetase/aspartate transcarbamylase*	urea cycle disorders, neonatal pulmonary hypertension	33	1500/2214
**Import**					
DNAJC19	*MDJ2*	*subunit of the mitochondrial import motor;*	cardiomyopathy, ataxia	30	116/146
GFER	*ERV1*	*oxidase of the disulfide relay system*	myopathy	26	205/189
HSPD1	*HSP60*	*mitochondrial chaperonin*	spastic paraplegia	55	573/572
TIMM8A	*TIM8*	*chaperone of the subunit Tim23 of the translocase of the inner mitochondrial membrane*	deafness, optic atrophy, Mohr-Tranebjaerg syndrome	33	97/87
MAGMAS (TIM16)	*PAM16*	*subunit of the translocase of the inner mitochondrial membrane*	spondylodysplastic dysplasia	35	125/149
**Proteases**					
SPG7	*AFG3*	*m-AAA metalloprotease*	spastic paraplegia	36	795/761
AFG3L2	*YTA12 (RCA1)*	*m-AAA metalloprotease*	spastic ataxia, neuropathy	44	797/825
**Fission/fusion**					
DNM1L-(DRP1)	*DNM1*	*dynamin-related GTPase mediating mitochondrial fission*	encephalopathy	43	736/757
MFN2	*FZO1*	*GTPase mediating OM fusion*	optic atrophy, neuropathy	14	757/855
OPA1	*MGM1*	*dynamin-related GTPase mediating IM fusion*	optic atrophy	17	960/881

H.s. genes	*S.c.* genes	Molecular function within mitochondria	Mitochondrial Diseases	% id	*H.s.* /*S.c.* protein length
Mitochondrial genes					
**Respiratory complex III**					
MT-CYTB	*COB (CYTB)*	*catalytic subunit of complex III, cytochrome b*	exercice intolerance, cardiomyopathy	48	380/385
**Respiratory complex IV**					
MT-COX1	*COX1*	*catalytic subunit of complex IV*	complex IV deficit, deafness, MELAS and Leigh syndromes, myoglobinuria	56	513/534
MT-COX2	*COX2*	*catalytic subunit of complex IV*	complex IV deficit, deafness, MELAS and Leigh syndromes, myoglobinuria	38	227/251
**ATP synthase**					
ATP6	*ATP6*	*Subunit a of the F0 sector of mitochondrial F1F0 ATP synthase*	NARP and Leigh syndromes, cardiopathy, spastic paraplegia	30	226/259
ATP8	*ATP8*	*Subunit A6L of the F0 sector of mitochondrial F1F0 ATP synthase*	Hypertrophic cardiomyopathy, Neuropathy	19	68/48

Table 1. Yeast homologs of human genes involved in mitochondrial disease

Databases used to compile this table: UniProt (http://www.uniprot.org/), CilDB (http://cildb.cgm.cnrs-gif.fr/), SGD (http://www.yeastgenome.org/) and ORFANET (http://www.orpha.net). All the listed nuclear genes encoded mitochondrial proteins except those marked by §, which are cytosolic proteins. The gene marked by * is absent in *S. cerevisiae* but present in *Schizosaccharomyces pombe*. *H.s., Homo sapiens; S.c., Saccharomyces cerevisiae*; % id, percentage identity.
